# Neoadjuvant anti-programmed Death-1 immunotherapy by Pembrolizumab in resectable nodal positive stage II/IIIa non-small-cell lung cancer (NSCLC): the NEOMUN trial

**DOI:** 10.1186/s12885-019-5624-2

**Published:** 2019-05-02

**Authors:** Florian Eichhorn, Laura V. Klotz, Helge Bischoff, Michael Thomas, Felix Lasitschka, Hauke Winter, Hans Hoffmann, Martin E. Eichhorn

**Affiliations:** 1 Department of Thoracic Surgery, Thoraxklinik, Heidelberg University, Roentgenstraße 1, 69126 Heidelberg, Germany; 2 Institute of Pathology, Heidelberg University, Heidelberg, Germany; 3 Divison of Thoracic Surgery, Technical University of Munich, Munich, Germany; 4 Translational Lung Research Center (TLRC), Member of German Center for Lung Research (DZL), Heidelberg, Germany; 5 Department of Thoracic Oncology, Thoraxklinik, Heidelberg University, Heidelberg, Germany

**Keywords:** NSCLC, Neoadjuvant immunotherapy, Checkpoint inhibitor, Thoracic surgery, Pembrolizumab

## Abstract

**Background:**

Immunotherapies targeting the PD1/PD-L1 pathway have had a large impact on the treatment of advanced NSCLC. Concerning multimodality tumor therapy, only few trials until today have been performed investigating neoadjuvant treatment with anti PD-1 immunotherapy prior to curative intent surgery. Aim of the NEOMUN investigator initiated trial (EudraCT-Number: 2017–000105-20; ClinicalTrials.gov Identifier: NCT03197467) is to assess feasibility and safety of pre-surgical anti PD-1 treatment in order to improve long term survival.

**Methods:**

The study is designed as an open-label, single arm, prospective, monocenter, phase II study including 30 patients with NSCLC stage II/IIIA suitable for curative intent surgery. Investigational drug is Pembrolizumab. After 2 cycles of immunotherapy (à 200 mg q3w i.v.), tumor resection with lobectomy or bilobectomy will be performed. Primary objectives are to assess the feasibility and safety of a neoadjuvant immunotherapy and to assess antitumor activity of Pembrolizumab with regard to clinical and pathological tumor response. Secondary objective is disease free and overall survival. Exploratory objective is to analyze potential predictive biomarkers and to evaluate the therapeutic efficacy of Pembrolizumab by extended immune cell and cytokine analysis of tumor tissue.

The study protocol was approved by the local ethics committee and the federal authority. Start of patient enrollment is scheduled for June 2018.

**Discussion:**

The NEOMUN trial will be one of the first clinical trials investigating a multimodal treatment strategy including neoadjuvant immunotherapy using Pembrolizumab as an investigational drug. Assessing the safety and therapeutic potential of neoadjuvant immunotherapy in connection with lung surgery will be of great interest for thoracic surgeons.

**Trial registration:**

Prospectively, the NEOMUN study has been registered on www.clinicaltrials.gov; NCT03197467 (first post: June 23rd, 2017).

## Background

Lung cancer is one of the leading causes of death worldwide with approximately 230.000 new diagnoses per year in the United States and 410.000 in Europe [1, 2]. Non-small cell histology (NSCLC) accounts for 80% of all lung cancer patients [3]. Less than two thirds of all patients with a newly diagnosed NSCLC have the chance of a curative treatment. Only about 25% are diagnosed in a very early stage (stages IA to IB) [4, 5]. In these early stages, 5-year-survival ranges from 60 to 80% after radical surgical treatment [6]. Patients with locally advanced (T2-T4) tumors or limited nodal disease (N1, unilevel N2) do profit from a multimodal therapy including surgery followed by adjuvant cisplatin duplet chemotherapy. Nevertheless, survival of patients in stages IIA and IIB with nodal tumor involvement (N1) is estimated between 55 and 35% at five years and is even worse (15 to 40%) for patients with locally resectable tumors but limited nodal N2-disease. Following complete microscopic resection (R0), adjuvant chemotherapy may improve survival benefit of only approximately 5% [7, 8]. Until now, the recommendation for adjuvant treatment in resectable stages IIA to IIIA NSCLC is based on a meta-analysis of few studies that have been conducted up to 20 years ago [9].

In contrast to standard cisplatin based chemotherapy, novel drugs modulating the patient’s immune-system unleashing the anti-tumor immunity have been implemented into modern treatment strategies. Special interest has been raised by so-called regulatory immune-inhibitory pathways including the programmed death-receptor 1 (PD-1) and its ligand (PD-L1). Besides others, these molecules are expressed on the tumor cell surface (PD-L1) or the patients activated T-cells (PD-1). Ligation of PD1/PD-L1 suppresses activated T-cells, thus resulting in impaired anti-tumor immunity [10, 11].

Antibodies blocking the ligation of PD-1/PD-L1 have been investigated in recent clinical trials in NSCLC stage IV patients (KEYNOTE-trials). Treatment of selected patients (PD-L1 expression on at least 50% of the tumor-cells, no EGF-receptor mutation or ALK-translocation) with a monoclonal humanized antibody against PD-1 (Pembrolizumab, KEYTRUDA©) correlated with an increased progression-free survival (median 10.3 months vs. 6.0 months, *p* < 0.001) and an overall survival of 80.2% at 6 months compared to 72.4% in patients receiving standard chemotherapy (*p* < 0.005). These findings resulted in the recent approval of Pembrolizumab as first line-treatment for patients with a PD-L1 expression > 50%. Nevertheless, the KEYNOTE study population consisted of patients with advanced or metastatic NSCLC [10].

The feasibility and safety of *neoadjuvant* immunotherapy prior to tumor resection has been proven in a recently published study by Forde et al. Preliminary data shows encouraging results in clinical and pathological response evaluation [12]. Based on these experiences, aim of the NEOMUN investigator initiated trial (NCT03197467; EudraCT No.: 2017–000105-20) is to assess feasibility and safety of neoadjuvant anti PD-1 immunotherapy followed by curative intent surgery. Monitoring clinical and radiological response will be supplemented by a translational interdisciplinary research program. Assessing the induction of tumor specific immunity will help to more intensively explore potential immunotherapy-associated changes in the tumor and its microenvironment.

## Methods

### Trial design

The study is designed as a mono-center, open-label, single arm, prospective, phase II study. Pembrolizumab (KEYTRUDA®) will be administered in a neoadjuvant setting to patients with resectable NSCLC stage II/IIIA who are eligible for curative intent surgery. The percentage of patients reaching the surgical therapy following neoadjuvant immunotherapy should exceed 80%.

### Objectives of the study

*Primary Objectives* are to assess the feasibility and safety of a neoadjuvant application of Pembrolizumab and to evaluate the effectivity of an anti-PD1-treatment on clinical and pathologic tumor response.

*Secondary Objective* is to assess the impact of neoadjuvant Pembrolizumab application on patient disease free- and overall survival.

*Exploratory objective* is the translational assessment of treated and untreated tumors (e.g. inflammatory infiltrates in and around the resected tumor, serum- and tumor tissue cytokine concentrations, multi-OMICS tissue analysis; see section “Translational research”) in order to generate a hypothesis on potential biomarkers predicting the efficacy of Pembrolizumab.

### Endpoints of the study

*Primary endpoints* are to evaluate the frequency and severity of adverse events including peri- and post-operative complications (grade 2–4 AEs according to NCI-CTCAE V4.03) in all participants. Immunotherapy-associated tumor response will be assessed by

- radiological change (Δ tumor size / lymph node size), according to RECIST [13] and iRECIST* ([14]).
- functional (PET-activity (standardized uptake value [SUV] [15]) and
- pathological response parameters (regression grading according to Junker criteria [16])

*Immunotherapy-trials described unique patterns of tumor response (pseudoprogression). A subset of patients meeting RECIST 1.1 criteria for disease progression (in size or number of lesions) showed delayed but durable responses to therapy by time. iRECIST therefore widens RECIST to immune-related response criteria, notably iUPD (unconfirmed progressive disease). A new or growing lesion is then defined as iUPD until definite progression is confirmed over time.

#### *Secondary endpoints are* disease free- and overall survival

### Sample size calculation

Targeted sample size is 30 patients. The rationale for the sample size is based on ethical, clinical and scientific considerations. The neoadjuvant treatment approach with an immune checkpoint inhibitor is experimental with only limited data on safety and feasibility available until today. Therefore, only a small number of patients should be subjected to this experimental treatment. The sample size should allow the generation of statistically meaningful evidence for feasibility and safety to permit the decision to further develop this treatment strategy. With a sample size of *N* = 30 and assuming that the number of events follows a binomial distribution B (30,p), events with an incidence rate *p* > 9.5% will be observed at least once with a 95% probability. This observation limit will exclude individual Pembrolizumab related SAEs but covers most of the historical reference events/rates (e.g. more frequently reported SAE/AE in Pembrolizumab monotherapy trials [e.g. pneumonia, pleural effusion, pneumonitis, dyspnea, pulmonary embolism], reported pathological and radiological response rates to Pembrolizumab treatment). The reported frequency of these events has been taken into account to calculate the 95%-confidence interval (under a sample size of *n* = 30) using the Clopper-Pearson-Method [17, 18].

### Patient selection/screening

All patients (male and female, age: 18 years and older, ECOG performance status 0–1) with clinically suspected lung cancer that are scheduled for surgical cancer therapy at the Thoraxklinik Heidelberg will be screened for eligibility to participate in the NEOMUN-trial. Baseline inclusion criteria are non-small cell histology in clinical stages IIA to IIIA.

The decision for radical surgical therapy as an integral part of the therapy must be agreed on the oncological interdisciplinary tumor board. Each possible candidate is simultaneously re-evaluated by the principal investigator or authorized medical staff. Only patients with adequate cardiopulmonary function according to current treatment guidelines will be counted eligible for trial inclusion [19]. Further inclusion criteria are displayed in Table 1.

**Table 1:** Inclusion and exclusion criteria for participation in the NEOMUN trial

*Key inclusion criteria:*	*Key exclusion criteria:*
Age ≥ 18 years, ECOG status 0–1	Active autoimmune disease
	History of chronic systemic steroid therapy
Histologically confirmed NSCLC	Anticancer treatment < 30 days
Clinical stage IIA-IIIA according to the TNM classification, 7th edition [20]: (stages IIIA_1–3*_ Robinson classification [36])	Clinical T4 tumor (according to the 7th edition of the TNM-system [20])
Staging by PET/CT and brain magnetic resonance imaging (MRI)	Status post or current pneumonitis that required steroids
At least 1 measurable lesion according to RECIST 1.1 [13]	Monoclonal antibody-treatment (< 30 days)
	Live vaccination (< 30 days)
	Targeted small molecule therapy
	Previous treatment with checkpoint inhibitors
Adequate bone marrow function, liver and renal function	Symptomatic acute cardiovascular or cerebrovascular disease
	Evidence of interstitial lung disease
Adequate lung and cardiac function for intended lung resection according to German S3 guideline [37]	Psychiatric illness that might affect the patient’s ability to understand the demands of the clinical trial
Note: subjects of childbearing potential must use an adequate method of contraception	History of allogeneic tissue/solid organ transplant
	Hypersensitivity to Pembrolizumab
	Known active TBC, HBV, HCV or HIV infection

A full list of inclusion/exclusion criteria on https://clinicaltrials.gov/ct2/show/NCT03197467 Protocol Version 3.0, October 16th, 2017

### Timeline

All patients with histologically proven NSCLC in stages IIA to IIIA who are likely to meet all of the inclusion criteria and none of the exclusion criteria, may be included into the trial between day − 28 and 0. The treatment period will start after the first administration of Pembrolizumab (day 1). The second application is planned at day 21. During the pre-surgical period between day 42 to 50, a second PET-scan will be performed for radiological baseline assessment followed by curative intent surgery (day 50+).

**Fig. 1:**
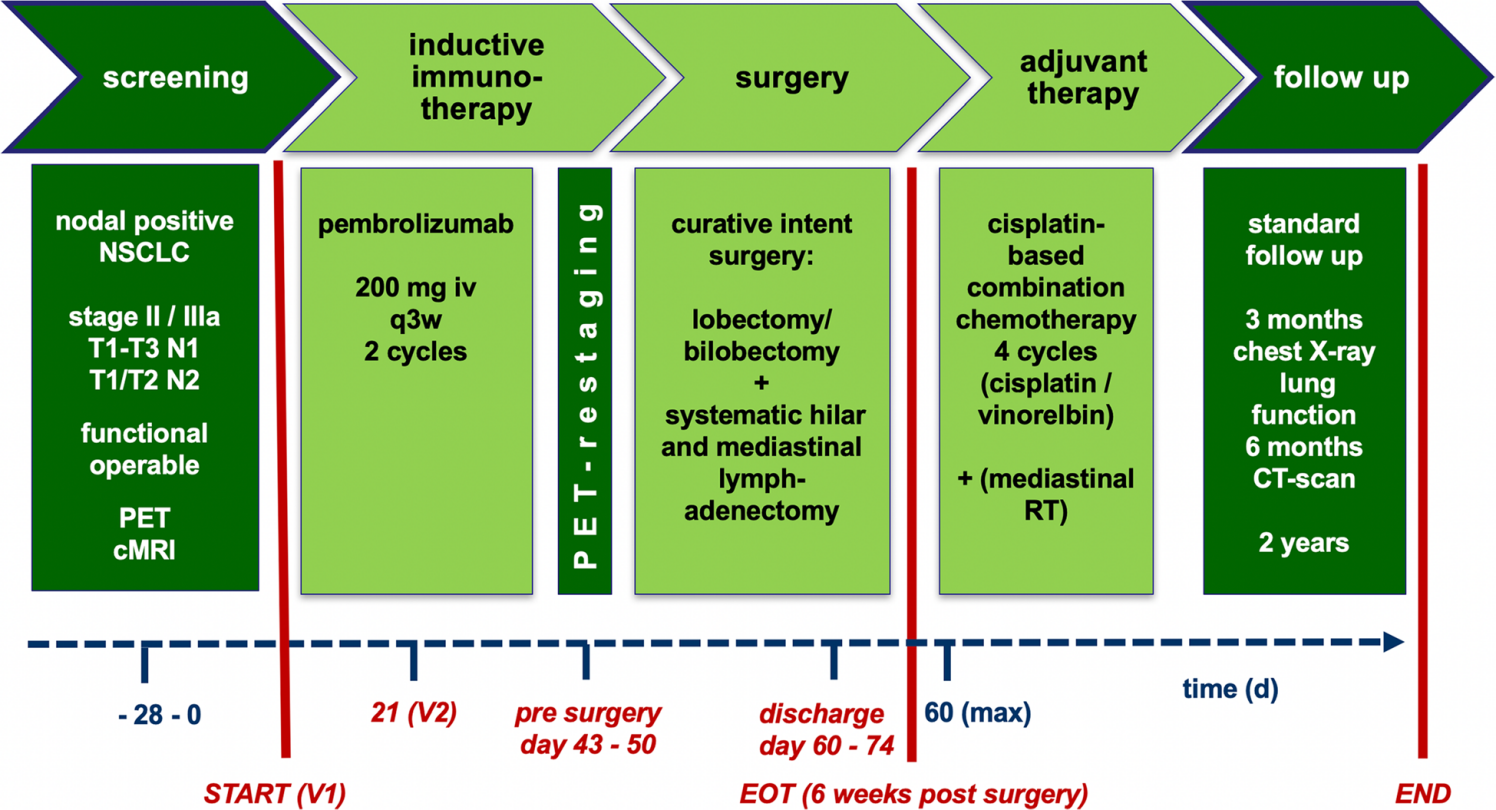
Timeline and summarized study protocol V1, V2: First and second administration of Pembrolizumab at day 0 and day (d) 21. Q3w: every three weeks; EOT: end of treatment; RT: radiotherapy; PET: positron emission tomography; iv: intravenous; d: day; cMRI: cranium Magnet Resonance Tomography

Adjuvant standard chemo- and/or radiotherapy will be administered according to the current guidelines. Each patient will be followed up 24 months after completion of the entire tumor therapy (Fig. 1).

### Procedures

#### Clinical staging/histological assessment

A contrast enhanced thoracic computed tomography (CT) will initially be performed in all patients that are screened for the trial. Distant metastatic disease will be radiologically ruled out using a diagnostic positron-emission-tomography-scan (PET) of the whole body and magnetic resonance imaging of the brain (MRI).

All patients receive rigid and flexible bronchoscopy to evaluate the endobronchial anatomy. The mediastinum is examined using endobronchial ultrasound. Enlarged (> 1 cm in conventional chest CT) or PET positive mediastinal lymph nodes will be clarified by EBUS-guided biopsy. If malignancy cannot be ruled out, mediastinoscopy will be performed. Histological confirmation of NSCLC will be provided by either transbronchial biopsy (TBB), transbronchial needle aspiration (TBNA) or transthoracic biopsy (ultrasound-guided or computed tomography guided).

Clinical staging will be proceeded in accordance to the actual TNM-classification. As defined by protocol, patients that meet the stage inclusion criteria according the 7th edition will be screened for eligibility to participate (Stages IIA - IIIA). Nevertheless, TNM-classification from the 7th and the actual valid 8th edition will both be provided [5, 20] .

#### Neoadjuvant immunotherapy

Investigational drug is intravenous Pembrolizumab at a fixed dose of 200 mg every three weeks ((q3w), 21 days) for 2 cycles (day 0, day 21).

#### Surgery

Following neoadjuvant immunotherapy, curative intent radical surgery will be performed in each patient meeting eligibility criteria. Thoracic surgery is no study procedure. Depending on individual patient factors (e.g. body weight, height, thoracic anatomy and tumor location), access into the chest cavity will be provided either by open surgery (anterolateral horizontal incision over the 3rd to 5th intercostal space) or endoscopic surgery (video-assisted thoracoscopy; one to three individually placed small utility incisions triangulated over the lateral chest wall). The study investigators will plan the patients for at least anatomical pulmonary lobectomy including systematic complete hilar and mediastinal lymphadenectomy, as systematic nodal dissection is an integral part of the surgical procedure to accurately assess the patients correct regional nodal status [21].

#### Adjuvant therapy

All patients will be discussed in the institutional multidisciplinary tumorboard before discharge. Depending on the individual tumor stage and postsurgical physical recovery, adjuvant chemotherapy will be recommended according to the current national and international guidelines.

### Long-term follow up

After completion of the initially recommended tumor-specific therapy, routine follow up will be performed every three months up to 24 months and includes thoracic and abdominal sectional imaging as well as lung function assessment. In addition, translational blood analysis will be conducted on e.g. tumor markers, peripheral blood mononuclear cells, antibody profiles and cellular as well humoral immune-related response parameters. Clinical or radiological findings suspicious for tumor recurrence will result in whole body restaging and further individual examination. The end of the entire trial is defined as the end of follow up of the last patient included.

### Adverse events

An adverse event (AE) is defined in the International Conference on Harmonization (ICH) Guideline for Good Clinical Practice as “any untoward medical occurrence in a patient or clinical investigation subject administered a pharmaceutical product and which does not necessarily have a causal relationship with this treatment” (ICH E6: section 1.2). An adverse event can therefore be any unfavorable and unintended sign (including an abnormal laboratory finding, for example), symptom, or disease temporally associated with the use of a medicinal (investigational) product, whether or not considered related to the medicinal (investigational) product.

A serious adverse event (SAE) is defined as any untoward medical occurrence (adverse event) that at any dose results in death, is life-threatening, requires new or prolonged in-patient hospitalization, results in persistent or significant disability/incapacity, is a congenital anomaly/birth defect or, any other significant medical condition.

Progression of the NSCLC and symptoms caused by progression of the NSCLC need not to be reported as SAE in this protocol, unless progression or symptoms of progression are assessed as causally related to study medication.

### Study discontinuation

The whole study will be stopped in the event of any of the following:

- Medical or ethical reasons affecting the continued performance of the study
- Occurrence of AEs unknown to date in respect of their nature, severity, and duration or the unexpected incidence of known AE with a negative impact on the risk/benefit assessment
- medically unacceptable risks for the treatment of the patients
- At the interim analysis (15 patients included): if the rate of unresectability is equal or greater 20%; i.e. if 3/15 or more study subjects did not undergo surgery as planned according to the protocol (unresectable disease or excessive delay of surgery).

### Translational research

All patients that are included into the trial will participate in a translational research program. The standard collection of specimen includes pre-therapeutic diagnostic biopsy, surgical specimen and sequential blood samples. Of major interest is the analysis of possible therapy-associated changes in different tumor regions (eg. Tumor core vs. marginal zone), the mediastinal and hilar lymph-nodes, the healthy lung tissue and the peripheral blood. Detailed research will be conducted to further investigate the tumor microenvironment, tumor mutational burden, mutational status, cytokine expression levels and genomic alterations. Further examinations of tumor tissue taken pre- and post Pembrolizumab will include whole genome sequencing and FACS-examination of the intratumoral immune infiltrate. Polarization of tumor associated macrophages (TAMs) in response to anti PD-1 treatment will be investigated.

Workup of pre- and posttreatment blood will focus on cytokine−/chemokine-profiles and antibody-profiles using multiplex assays. Specific interactions between tumor and immune-cells will be examined by further research on neo-epitope- specific T-cell responses and T-cell-receptor -clonality analysis. In order to evaluate a correlation between the mutational-load/transcriptome of the tumor and the peripheral blood, whole exome sequencing of cell free plasma DNA will be performed.

Data of the patients participating in the NEOMUN trial will be compared to patient data from an institutional biobank cohort matched to age, gender, stage of disease and tumor-dependent parameters (histology, size, nodal status). The matched cohort will only include patients who did not receive immunomodulatory treatment.

### Data analysis

All demographic and clinical characteristics recorded at baseline will be submitted to descriptive analyses using descriptive statistics by means of listing and tables. Continuous data will be summarized with at least the following: frequency (n), median, quartiles, mean, standard deviation (standard error), minimum and maximum. Statistical test between changes of parameters over time will be performed for continuous as well as categorical variables. Wilcoxon signed rank test, Fisher’s exact test and other appropriate tests will be used where applicable. Time-to-event measures (OS, DFS) will be analyzed by means of Kaplan-Meier curves. A *p*-value of < 0.05 will be considered statistically significant.

For the analysis of adverse events, summary tables providing the number and percentage of patients will be generated for the incidence of AEs overall and by severity. Laboratory parameters will be reported with descriptive statistics.

## Discussion

Patients with NSCLC and nodal N1 and/or N2 disease suffer from an overall relapse rate of 52% even when multimodality treatment including curative intent surgery with chemo- and/or radiotherapy is administered. The overall 5-year survival benefit in these patients accounts of only 4 to 5% [22, 23]. In terms of efficacy, no difference between a pre- or postoperative chemotherapy approach can be determined [24]. In the light of the efficacy results it is clear, that new multimodal treatment strategies are direly needed in order to improve long term survival following curative intent surgery.

Recently, immunotherapies targeting the PD-1/PD-L1 pathway have had significant impact on survival in patients with advanced stages of NSCLC in comparison to standard chemotherapy [10]. A correlation between PD-L1-expression (> 50%) and survival or response to immunotherapy has been shown in recent anti-PD-1 trials [25, 26]. However, the efficacy of Pembrolizumab as a first-line treatment in advanced and/or metastatic NSCLC patients with PD-L1 expression below 50% is under debate. Based upon preliminary promising results from the KEYNOTE-trials, the combination of Pembrolizumab and pemetrexed/carboplatin chemotherapy has been recently approved as first-line treatment for metastatic or advanced NSCLC regardless of the PD-L1 expression [27]. Further results are eagerly awaited to define more subgroups that are suitable for anti-PD-1 therapy.

Irrespective of the results in metastatic stage IV patients, the predictive value of the PD-L1 status might have a different impact in patients with non-metastatic earlier-stage lung cancer with less tumor burden. This hypothesis is supported by data recently published by Forde et al. [12]. Their trial (NCT02259621) investigated safety and feasibility of patients with early stage I-IIIa NSCLC treated neoadjuvantly with 2 doses of nivolumab. Major pathologic tumor response (defined as less than 10% viable tumor cells posttreatment) was observed in 9 of 21 (43%) resected tumors. Importantly, this tumor response did not correlate with the pre-treatment PD-L1 status assessed in tumor biopsies by immunohistochemistry.

Moreover, the techniques used to quantitatively assess the PDL-1 status in small pre-treatment biopsies are under critical debate. Several factors contributing to a false-negative or a false-low PD-L1 status have recently been identified [28]. From a biological point of view dynamic induction of PD-L1 expression and an intratumoral heterogeneity of PDL-1 expression may result in a false-negative PD-L1 status and may not be accurately investigated by small sample biopsies. This is supported by a comparative study investigating the PD-L1 status in surgically resected specimens and matched biopsies of NSCLC patients. The study revealed major discordances between biopsies and surgical specimens with an overall discordance rate of 48%. In all cases the biopsy specimens underestimated the PDL-1 status observed on the whole tissue sample [28]. Due to the points listed above, the authors believe that the benefit/risk ratio is not influenced by the pretreatment PDL-1 status and therefore the inclusion of an all-comers population is justifiable. The translational study research program will raise the opportunity to generate further data to elucidate the role of different PD-L1 expression levels in the context of neoadjuvant immunotherapy.

In addition to the PD-L1 status analysis, EGFR-mutation and ALK-translocation have been implemented in routine diagnostics and represent important prognostic parameters in the era of targeted therapy [29]. A correlation between positive EGFR-mutation and high PD-L1 expression in a historical cohort analysis of surgically resected NSCLC has been shown by Azuma et al. [30]. Several authors have also reported about rare concomitant expression of EGFR, ALK and/or PD-L1 and thus they so far refuted their mutual exclusiveness [31, 32]. Nevertheless, the NEOMUN-trial aims to include untreated patients with stage II/IIIA1–3 NSCLC. According to current guidelines surgical tumor resection is the primary treatment in this patient cohort. This also accounts for EGFR or ALK positive tumors. To date, there is no neoadjuvant or adjuvant EGFR- or ALK-TKI therapy authorized in this patient cohort. Based on this, the protocol does not include stratification after EGFR/ALK- mutation analysis and therefore does not exclude patients with positive mutations.

The question whether checkpoint blockade should be performed before or after surgery has been addressed by Liu et al. in a preclinical breast-cancer model. The authors reported about an increased tumor specific CD8+ T-cell response in patients after neoadjuvant immunotherapy [33]. Pircher et al. observed a reduction of regulatory T-cells (Tregs) in NSCLC patients that received neoadjuvant immune-chemotherapy (Cetuximab/Docetaxel/Cisplatin) before surgical resection [34]. Tregs play a relevant role in cancer-associated immune-escape mechanisms as they suppress cytotoxic T-lymphocytes and natural killer cells [35]. Taking this into account, it is uncontroversial that a profound interaction between the tumor and its autologous immune-system is necessary to induce tumorspecific t-cells to overcome tumor induced tolerance and to generate and activate T-cell memory. We hypothesize that the anti-neoplastic immune-stimulatory effect will be more effective in patients treated prior to tumor resection. Therefore, neoadjuvant immunotherapy by administration of anti-PD-1 targeting antibodies is very promising and has led to unforeseen therapeutic efficacy. The results of a number of ongoing clinical phase II and III trials addressing neoadjuvant immunotherapy before surgery in extrathoracic solid cancers are awaited with great interest (e.g. bladder cancer, melanoma, clinicaltrials.gov).

In NSCLC, clinical data on neoadjuvant immunomodulatory treatment is scarce but few encouraging trials investigating anti-PD-1/PD-L1 checkpoint inhibition prior to surgery for NSCLC patients are currently ongoing (e.g. NCT02938624; NCT03217071; NCT02818920; NCT02259621). In a first interim analysis, Forde et al. recently published data on patients with NSCLC receiving neoadjuvant anti-PD-1 therapy (Nivolumab). They observed a major pathological response (less than 10% viable tumor cells) in 9 of 20 patients (45%) with neoadjuvant immunotherapy. These preliminary data and the results of the KEYNOTE-trials identified immunotherapy as a promising strategy in NSCLC treatment [10].

The scope of the enrolled NEOMUN trial is to generate pilot data on the efficacy, safety and the feasibility of a neoadjuvant anti-PD-1 immunotherapy with Pembrolizumab. The research endeavor will be flanked by a translational research program to elucidate potential predictive biomarkers and to investigate the mode of action of anti PD-1 treatment in NSCLC patients.

## Abbreviations

AE: Adverse event
ALK: Anaplastic lymphoma kinase
CD: Cluster of differentiation
CT: Computed tomography
DFS: Disease free survival
DNA: Deoxyribonucleic acid
EBUS: Endobronchial ultrasound
ECOG: Eastern Cooperative Oncology Group
EGF: Epithelial growth factor
FACS: Fluorescence-activated cell scanning
HBV: Hepatitis b virus
HCV: Hepatitis c virus
HIV: Human immunodeficiency virus
ICH: International Conference on Harmonization
iRECIST: (RECIST for immune-based therapeutics)
MRI: Magnetic resonance imaging
NCI-CTCAE: National Cancer Institute Common Terminology Criteria for Adverse Events
NSCLC: Non-small cell lung cancer
OS: Overall survival
PD1/PD-L1: Programmed death 1 receptor/−ligand
PET: Position emission tomography
Q3w: Every three weeks
RECIST: Response Evaluation Criteria In Solid Tumors
SAE: Serious adverse event
SUV: Standard uptake value
TBB: Transbronchial biopsy
TBC: Tuberculosis
TBNA: Transbronchial needle aspiration
TNM: Tumor node metastasis
Tregs: Regulatory T-cell
